# Deciphering the Endophytic and Rhizospheric Microbial Communities of a Metallophyte *Commelina communis* in Different Cu-Polluted Soils

**DOI:** 10.3390/microorganisms9081689

**Published:** 2021-08-09

**Authors:** Li He, Yanzhen Ren, Weimin Zeng, Xueling Wu, Li Shen, Runlan Yu, Yuandong Liu, Jiaokun Li

**Affiliations:** 1 School of Minerals Processing and Bioengineering, Central South University, Changsha 410083, China; 195612132@csu.edu.cn (L.H.); 185612135@csu.edu.cn (Y.R.); zengweimin1024@126.com (W.Z.); wxlcsu@csu.edu.cn (X.W.); lishen@csu.edu.cn (L.S.); yrl715@sina.com (R.Y.); ydoliu@sina.cn (Y.L.); 2 Key Laboratory of Biometallurgy, Ministry of Education, Central South University (CSU), Changsha 410083, China

**Keywords:** high-throughput sequencing, bacterial community, *Commelina communis*, endophytic bacteria, rhizosphere bacteria

## Abstract

Metallophytes microbiota play a key role in plant growth and resistance to heavy metal stress. Comparing to the well-studied single or some specific plant growth-promoting (PGP) bacterial strains, our current understanding of the structural and functional variations of microbiome of metallophytes is still limited. Here, we systematically investigated the endophytic and rhizosphere bacterial community profiles of a metallophyte *Commelina communis* growing in different Cu-polluted soils by high-throughput sequencing technology. The results showed that the rhizosphere communities of *C*. *communis* exhibited a much higher level of diversity and richness than the endosphere communities. Meanwhile, shifts in the bacterial community composition were observed between the rhizosphere and endosphere of *C*. *communis*, indicating plant compartment was a strong driver for the divergence between rhizosphere and endosphere community. Among the environmental factors, soil Cu content, followed by OM, TP and TN, played major roles in shaping the bacterial community structure of *C*. *communis*. At the highly Cu-contaminated site, *Pseudomonas* and *Sphingomonas* were the predominant genera in the endophytic and rhizospheric bacterial communities, respectively, which might enhance copper tolerance as PGP bacteria. In summary, our findings will be useful to better understand metallophyte–microbe interactions and select suitable bacterial taxa when facilitating phytoremediation.

## 1. Introduction

Heavy metals in soil pose a serious threat to the entire ecosystem due to their high toxicity and non-biodegradable characteristics. The elevated concentrations of heavy metals are continuously entering into the food chain through agriculture, leading to considerable health risks to humans and animals; therefore, this issue requires urgent remediation [1,2]. Compared to other conventional physico-chemical remediation methods, phytoremediation has been considered as an alternative cost-effective and eco-friendly remediation strategy for *in situ* toxic-metal cleanup, attracting a lot of attention [3]. Although phytoremediation is a promising green technology, the application of phytoremediation is limited due to its low efficiency, which is caused by the limited biomass and heavy metal accumulation capacity. High excessive concentrations of metal ions in the soil can significantly induce oxidative damages, reduce the photosynthetic rate, and inhibit the plant growth and development [3]. 

Recent evidence indicates that the growth of metallophytes and tolerance to toxic metals may be connected to the beneficial effects of their endophytic and rhizosphere microorganisms [4,5]. Similar to metallophytes, these microorganisms can adapt to the extreme environments and directly or indirectly aid host plants in coping with metal-induced stress via different plant-growth-promoting activities, such as the secretion of indole-3-acetic acid (IAA), siderophores and 1-aminocyclopropane-1-carboxylic acid deaminase (ACCD), as well as nitrogen fixation and phosphorus solubilization [6,7]. Meanwhile, the bioavailability and phytotoxicity of heavy metals could also be potentially altered by these plant-associated microorganisms through releasing chelating agents, acidification, phosphate solubilization or redox changes [8,9]. To date, most of this research was based on traditional culture-dependent techniques and mainly focused on the impact of a single or some specific bacterial strains under laboratory and green house conditions; however, few studies have evaluated the structure and function of microbial communities in the endosphere and rhizosphere of metallophytes [10]. Consequently, we have limited knowledge on the interactions between microbes and plants at community level on native plant vegetation growing in metal-polluted environments [11]. 

It is believed that plant species is one of the main factors determining the composition of a soil bacterial community [12,13]. Plants can secrete not only the primary metabolites (mainly sugars, amino acids and organic acids), but also a variety of secondary metabolites into the rhizosphere, which can attract and shape the structure and function of microbial communities [14]. Besides, many abiotic factors such as soil type [15], organic matter [16], pH [17], oxidation-reduction potential [18] and metal ions [19] can also influence the composition and activity of microbial communities. Currently, however, the factors influencing the composition and structure of microbial communities in metallophytes still remain largely unknown. Meanwhile, although the microbiome inhabiting rhizosphere of metallophytes is a source of formation of the community of endophytic bacteria [13], we still lack the fundamental information concerning niche differentiation in their community structure and co-occurrence patterns associated with the rhizosphere microbial community.

*Commelina communis*, also known as dayflower, is a typical facultative metallophyte, which widely distributes on copper-contaminated soils and non-cupriferous habitats in China [20,21]. In previous studies, *C*. *communis* has been reported to be used in phytoremediation of copper-contaminated soils in China. Although some Cu-resistant endophytic and rhizospheric bacteria have been isolated from *C*. *communis* [22,23], there are few studies on the microbial communities associated with *C*. *communis*. To fill this gap, we systematically investigated the endophytic and rhizospheric microbiome of *C*. *communis* growing on different Cu-polluted soils through high-throughput sequencing technology. Specific questions we address include: (1) what is the structure and composition of endosphere and rhizosphere microbial communities in *C*. *communis*? (2) How do the endophytic and rhizospheric microbial communities of *C*. *communis* vary along a Cu contamination gradient? Additionally, (3) what are the key drivers if such variations exist? Our results provide a comprehensive insight into the complex bacterial community associated with metallophytes, which may be crucial for further application of the beneficial bacteria in phytoremediation. 

## 2. Materials and Methods

### 2.1. Plant and Soil Sample Collection 

Naturally growing *C*. *communis* were collected from one non-cupriferous and three separate copper-contaminated areas in the central part of China in July 2019 (Figure S1). The sample collection in the contaminated mining areas and normal soils was permitted by local producers and residents. The non-cupriferous site is located at Yuelu Mountain near the Central South University, in Changsha city (CS), Hunan Province, China (112°09′ E, 28°17′ N). Among the three cupriferous sites, two are located at the Qibaoshan (113°94′ E, 28°28′ N) and Longshang mining area (113°71′ E, 26°79′ N) in Liuyang city (LY) and Zhuzhou city (ZZ), respectively, Hunan Province, China. Another copper-contaminated site is located in the Tonglushan Copper Mine area, in Daye city (DY), Hubei Province, China (114°95′ E, 30°08′ N), which is one of the biggest copper production bases in China [24]. During the sampling period, all plants were in the flowering stage. Three healthy plants were randomly collected from each site and the whole plant with the surrounding soil (∼25 cm in length, ∼25 cm wide, and 15∼25 cm in depth) was excavated and brought to the laboratory within 24 h. 

### 2.2. Soil Properties Analysis

The air-dried soil samples were passed through a 2 mm screen sieve and analyzed for pH, electrical conductivity (EC), organic matter (OM), total nitrogen (TN), total phosphorus (TP), total potassium (TK), Calcium (Ca), Magnesium (Mg) and Copper (Cu) according to Sun et al. [25]. Briefly, pH and EC was measured with a soil-to-water ratio of 1:2.5 and 1:5 (*w*/*v*) using a pH meter (SG2, Mettler Toledo Instruments Co. Ltd., Shanghai, China) and EC meter (D-54, Horiba, Kyoto, Japan), respectively. OM was measured by the method of potassium dichromate oxidation heating after the soils were digested with H_2_SO_4_. TN and TP were determined by the micro-Kjeldahl and molybdenum antimony colorimetry method, respectively. TK was analyzed by flame photometry after digestion with sodium hydroxide. The contents of Ca and Mg were determined by the ammonium-acetate (1 N, pH 7.0) infusion method [26]. The Cu content was analyzed by inductively coupled plasma optical emission spectrometry (ICP-OES) (Optima 2000 DV, PerkinElmer, Shelton, CT, USA) after H_2_O_2_-HNO_3_ -HCl (1:4:2, *v*/*v*) digestion.

### 2.3. Endosphere and Rhizosphere Sample Preparation

Fractionation of the rhizosphere and the endosphere microbes was performed according to the methods of Dong et al. [27]. To recover the rhizosphere microorganisms, soils loosely adhered to the roots were firstly removed by violent shaking. The collected root samples were firstly washed with sterilized PBS buffer (Na_2_HPO_4_ 1.42 g/L; KH_2_ PO_4_ 0.24 g/L; KCl 0.2 g/L; NaCl 8 g/L; 0.01% Triton X-100, pH7.4) with shaking at 150 rpm for 30 min. Then, the soil suspension was centrifuged at 4000× *g* at 4 °C for 20 min and the soil pellets were defined as rhizospheric samples. Meanwhile, the plant materials (leaf, stem, and root) were collected and used for endophytic bacteria recovery. In order to remove the excess soils and epiphytic microorganisms, the plant samples were firstly washed with flowing tap water and then disinfected by placing them in 80% ethanol for 2 min before being treated with 5% sodium hypochlorite (NaClO). Finally, the plant samples were rinsed four times with sterile water. About 0.1 mL of the final wash was spread on LB plates and incubated overnight at 30 °C to check for contamination. After grinding with a sterilized mortar and pestle, the plant tissues were incubated in PBS buffer for 2 h, filtrated with four layers of gauze and centrifuged at 4000× *g* for 15 min to harvest the endophytes. The collected endosphere and rhizosphere microbes were frozen in liquid nitrogen (30 s), and stored at −80 °C before DNA extraction.

### 2.4. DNA Extraction and 16S rRNA Sequencing 

According to the manufacturer’s guidelines, the total DNA of the soil samples and plant materials were extracted using a Power Soil Extraction Kit (Mo Bio Laboratories, San Diego, CA, USA) and a DNeasy Plant Mini Kit (Qiagen, Hilden, Germany), respectively. The DNA concentration and quality were tested with an ND 2000 spectrophotometer (Thermo Scientific, Wilmington, DE, USA) and agarose (1%) gel electrophoresis. The primers 799F (5′-AACMGGATTAGATACCCKG-3′) and 1193R (5′-ACGTCATCCCCACCTTCC-3′) targeting the hypervariable V5-V7 regions were selected to characterize the diversity of the bacterial communities, which were proved useful to minimize the contamination of plant-derived sequences [28]. All PCRs were carried out in a 20 μL mixture containing 10 ng of template DNA, 0.8 μL of each primer (5 μM), 4 μL of 5 × FastPfu buffer, 2 μL of 2.5 mM dNTPs and 0.4 μL of FastPfu DNA Polymerase. The PCR reaction conditions were performed as follows: initial denaturation at 95 °C for 1 min; denaturing at 95 °C for 30 s; annealing at 55 °C for 30 s; extension at 72 °C for 45 s; 27 cycles; a final extension at 72 °C for 10 min. The amplified PCR products were recovered by 2% agarose gel, purified by the AxyPrep DNA Gel Extraction Kit (Axygen Biosciences, Union City, CA, USA) and further quantified by using Quantus™ Fluorometer (Promega, Madison, WI, USA) according to manufacturer’s instructions. Purified amplicons were subsequently sequenced on an Illumina MiSeq PE300 platform (Illumina, San Diego, CA, USA) by Majorbio Bio-Pharm Technology Co. Ltd. (Shanghai, China).

### 2.5. Bioinformatic Analyses

The raw sequencing reads were de-multiplexed, quality-filtered by fastp version 0.20.0 [29] and merged by FLASH version 1.2.7 [30]. The chimeric sequences as well as chloroplast and mitochondrial sequences were removed. The clean sequences were clustered into operational taxonomic units (OTUs) at a 97% similarity level using UPARSE version 7.1 [31]. The taxonomy of each bacterial sequence was analyzed by RDP Classifier version 2.2 [32] against the 16S rRNA database (Silva v138) with a 70% confidence threshold. To eliminate the effects of sequence number variation from different samples, we rarefied each sample to the minimum sequencing depth (7387 sequences per sample). The rarefaction analysis, alpha diversity indices, including Shannon index, Chao index, and Good’s coverage were conducted using Mothur v.1.30.1. The principal coordinate analysis (PCoA) and hierarchical clustering analysis based on Bray–Curtis distance were performed using R (http://www.r-project.org/, accessed on 1 December 2020). The Venn diagram was generated to visualize the shared and unique OTUs among groups based on the occurrence of OTUs across groups regardless of their relative abundances. The heat map was generated by the gplots package in R to show the proportion of the top 30 most abundant genera for each sample. The phylogenetic tree was generated using MEGA-X (neighbor-joining method, 1000 replicates) and the Interactive Tree of Life (iTOL, http://itol.embl.de, accessed on 1 December 2020) was used to visualize the core microbiomes. In addition, the linear discriminant analysis (LDA) effect size (LEfSe) method was performed to identify bacterial taxa with significantly different abundances between groups [33]. The Kruskal–Wallis (KW) sum-rank test (α = 0.05) was used in the LEfSe analysis to detect features with significantly different abundances between the specified categories, and this was followed by an LDA to estimate the effect size of each differentially abundant feature (logarithmic LDA score > 4.0). To investigate the effects of soil environmental factors on the bacterial structure and composition of *C*. *communis*, canonical correspondence analysis (CCA) was conducted using CANOCO 5.0 software. A network analysis was performed by using Networkx software [34]. Only Spearman correlations with an r > 0.6 (*p* < 0.05) were considered to indicate a valid interactive event.

### 2.6. Statistical Analyses 

All statistical analyses were performed on SPSS 22.0 (SPSS Inc., Chicago, IL, USA). Normal distributions of the data were checked with the Shapiro–Wilk test and homoscedasticity of variances was analyzed using Levene’s test. Significant differences in the variance of parameters were evaluated, depending on the distribution of the estimated parameters, either with one-way analysis of variance (ANOVA) or the Kruskal–Wallis rank sum test. Post hoc comparisons were conducted by either the Tukey’s honest significant differences test or Nemenyi test. The graphs and charts were generated by Origin 9.0 or R v. 3.4.0. 

## 3. Results

### 3.1. Physico-Chemical Properties of Rhizosphere Soils

The physico-chemical properties of triplicate rhizosphere soils were summarized in Table S1. We found that the contents of Cu in rhizospheric soils from DY (13,100 ± 132.28 mg/kg) and ZZ (248 ± 28.91 mg/kg) were far above their soil limit of the international primary standard (35 mg/kg) (GB15618-1995), while soils from LY (41 ± 3.61) showed relatively lower Cu contamination. As expected, the Cu concentration in soils from uncontaminated site (CS) (25 ± 3.15) was below the GB15618-1995 limit. Meanwhile, the soil samples from the Cu contaminated areas (LY, ZZ, and DY) showed significantly elevated pH, EC and higher contents of Mg and Ca compared with that from CS (*p* < 0.05). In CS, the rhizospheric soil of *C*. *communis* was slightly acidic (6.26), while in the contaminated areas, the pH values varied from slightly alkaline (7.37) in LY to alkaline in DY (8.16) and ZZ (8.31) (Table S1). The electrical conductivity of rhizospheric soils from Cu contaminated areas was relatively high, ranging from 278 ± 10.01 μS/cm to 952 ± 30.12 μS/cm, indicating a high salinity in these sites. In contrast, the contaminated sites except DY showed very low contents of OM, TN and TP (Table S1). 

### 3.2. General Features of the Sequencing Data

After quality control, a total of 689,659 and 263,895 high-quality sequences were obtained from the endophytic and rhizospheric samples, with an average read length of about 375.3 and 375.7 bp, respectively (Table S2). At a cut-off of 97% sequence similarity, a total of 3223 bacterial OTUs were identified and the number of OTUs per sample varied from 45 to 1086 (Table S2). The high Good’s coverage values (≥97%) indicated that the bacterial OTUs were well captured in each sample (Table S2). The rarefaction curves of the Shannon index tended to approach the saturation plateau with the increasing number of reads, thus indicating that the sequencing depth was sufficient and all the data were reasonable in this study (Figure S2). 

### 3.3. Diversity and Structure of the Endosphere and Rhizosphere Bacteria Community

**Figure 1:**
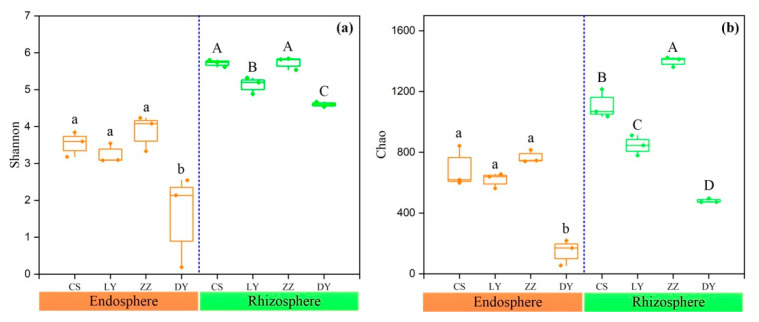
Alpha diversity of endosphere and rhizosphere bacterial microbial communities of *C*. *communis* at the four sampling sites. (**a**) Shannon’s diversity, (**b**) Chao richness. The horizontal bands within boxes represent the median and whiskers represent the minimum and maximum values. Data are analyzed by means of one-way ANOVAs and different small letters on the bars indicate significant differences at *p* < 0.05.

Alpha diversity, the microbial diversity within each sample, was analyzed based on the Shannon and Chao index (Figure 1). To control for differences in sampling effort across plant compartments, we rarefied each sample to 7387 sequences per sample before calculating the diversity indices. As shown in Figure S3, The Shannon and Chao estimator for the rhizosphere soil community was 1.71 times and 1.65 times higher than those in the endosphere communities. For the endophytic bacteria, the diversity and richness were lowest in the samples collected from DY (Figure 1a). Similarly, the Shannon and Chao indices of the rhizospheric bacteria in DY were also significantly lower than those in the other three sampling sites (Figure 1b). 

**Figure 2:**
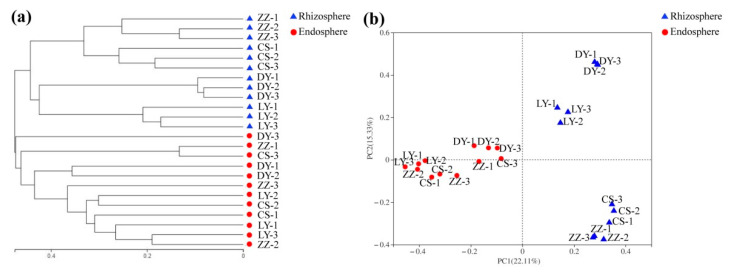
Microbial community differentiation in the endosphere and rhizosphere of *C*. *communis*. (**a**) Hierarchical clustering of the bacterial communities based on the Bray–Curtis dissimilarity of all of the samples. (**b**) Principal coordinate analysis (PCoA) of the bacterial communities based on the Bray–Curtis distances between samples.

In order to compare the microbial community composition of endosphere and rhizosphere among different sampling sites, hierarchical clustering was performed based on Bray–Curtis dissimilarities on normalized data at the OTU level. As shown in Figure 2a, microbial communities clearly differentiated between the endosphere and rhizosphere in all the samples. Meanwhile, this affinity relationship was also demonstrated by the PCoA results. As shown in Figure 2b, all the samples were clearly clustered into two groups: endosphere group and rhizosphere group. The results of ADONIS (*R*^2^ = 0.204, *p* = 0.001) and ANOSIM (*R* = 0.746, *p* = 0.001) testing based on Bray–Curtis dissimilarity confirmed the significant differences in the microbial composition between rhizosphere and endosphere of *C*. *communis*. Additionally, the endosphere bacteria from CS, LY and ZZ clustered together closely, indicating that these bacterial communities were very similar (Figure S4a). Similarly, in the rhizosphere of *C*. *communis*, the microorganisms from the DY were relatively distinct from the others (Figure S4b).

### 3.4. Venn Diagram for Rhizospheric and Endophytic Bacterial Communities

**Figure 3:**
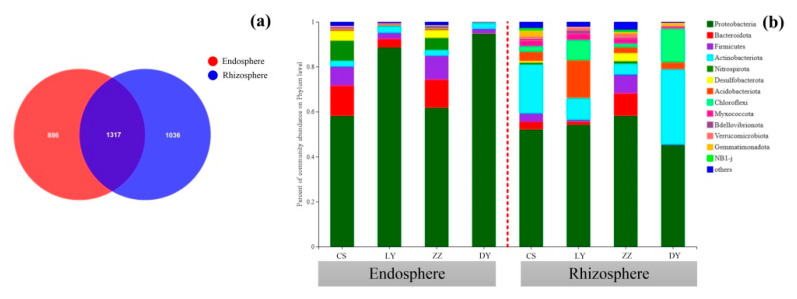
(**a**) Venn diagram of exclusive and shared bacterial OTUs (at the 3% evolutionary distance) between the endosphere and rhizosphere of *C*. *communis*. (**b**) Bacterial community composition at the phylum level in the endosphere and rhizosphere of *C*. *communis* in four different sampling sites.

The Venn diagram showed that the majority of bacterial OTUs identified in the root rhizosphere of *C*. *communis* were also present in the endophytic compartment (Figure 3a). A total of 3239 OTUs existed in endosphere and rhizosphere of *C*. *communis*, while 886 OTUs were only enriched in endosphere and 1036 OTUs in rhizosphere (Figure 3a). Moreover, there were 356, 258, 420 and 60 unique OTUs for endosphere samples of CS, LY, ZZ and DY, respectively, except for 119 core OTUs (Figure S5a). Similar results were also obtained in rhizosphere of *C*. *communis*. All rhizosphere samples shared 175 core OTUs with 214, 337, 545 and 131 OTUs being unique in the CS, LY, ZZ and DY, respectively (Figure S5b).

### 3.5. Taxonomic Distributions of the Endosphere and Rhizosphere Bacteria Community

In this study, 6 and 13 dominant bacterial phyla (relative abundance of >1% in at least in one group) were identified for endosphere and rhizosphere of *C*. *communis*, respectively. As shown in Figure S6, Proteobacteria (73.41%), Bacteroidota (8.46%) and Firmicutes (6.44%) were the three most abundant bacterial phyla in the endosphere. Meanwhile, the three most dominant bacterial phyla in the endosphere for CS, LY and ZZ samples were Proteobacteria (58.2%, 88.6% and 61.8%), Bacteroidota (13.4%, 3.7% and 12.5%) and Firmicutes (8.4%, 2.8% and 10.6%), respectively, whereas nearly all endosphere bacterial sequences belonged to Proteobacteria (94.7%) for DY samples (Figure 3b). For the rhizosphere microbial community, the most abundant phyla were Proteobacteria (ranging from 45.1% to 58.3%) and Actinobacteriota (ranging from 4.9 % to 33.4%), followed by Acidobacteriota (ranging from 2.6% to 16.7%) and Chloroflexi (ranging from 1.5% to 14.9%) (Figure 3b). Notably, the phyla Actinobacteriota and Chloroflexi were more abundant in rhizospheric samples in DY than the other three sampling sites (Figure 3b). 

**Figure 4:**
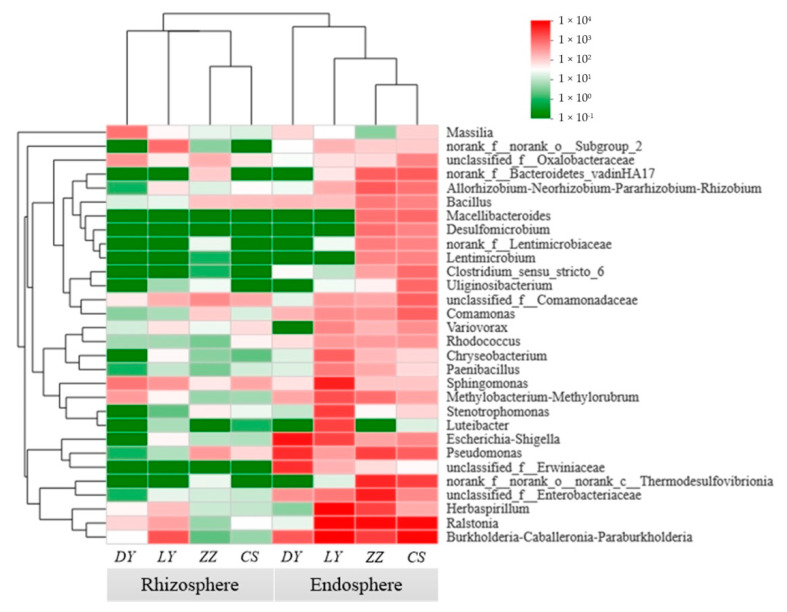
Heat map analysis of the dominant genera (top 30) distribution in the endosphere and rhizosphere of *C*. *communis.* The abundance is expressed as the color intensity, which reflects the proportion of the total effective sequences in each group.

Furthermore, the top 30 dominant microbial genera were selected and analyzed by a hierarchically clustered heat map analysis (Figure 4). At genus level, the most abundant genera in the *C*. *communis* endosphere were *Ralstonia* (14.69%), *Burkholderia–Caballeronia–Paraburkholderia* (13.58%), *Herbaspirillum* (7.18%), followed by *Pseudomonas* (4.51%) and *Sphingomonas* (3.05%). Compared to the endosphere, the bacterial communities in *C*. *communis* rhizosphere were predominated by *Burkholderia–Caballeronia–Paraburkholderia* (5.51%) and *Sphingomonas* (4.53%). In addition, the results also revealed that the bacterial community of *C*. *communis* could be split into two clusters. One cluster was mainly composed of endophytes samples, and the other one primarily comprised rhizosphere samples (Figure 4). In addition, the heat map also showed that there were differences in the relative abundances of bacterial genera among the four sampling sites (Figure 4). The composition of the endosphere and rhizosphere bacterial communities in the CS, LY and ZZ samples appeared to be more similar and more closely clustered together than those detected in DY samples (Figure 4). 

### 3.6. Phylogenetic Tree of the Core Microorganisms

**Figure 5:**
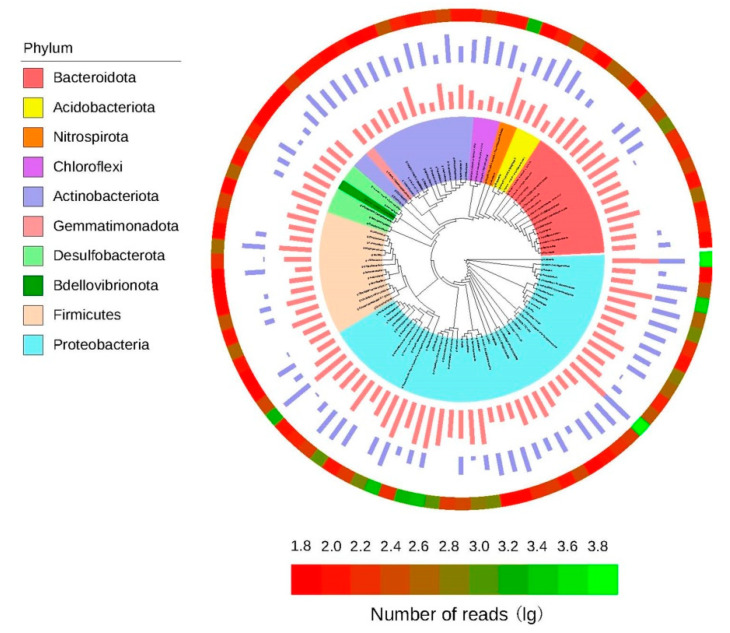
Phylogenetic characteristics of the endosphere and rhizosphere bacterial communities of *C*. *communis* (results visualized using iTOL tool). The phylogenetic tree is presented at the genus level and colored by phylum. The length of each bar represents the normalized mean relative abundance of a genus; a red bar indicates endosphere samples, and a purple bar indicates rhizosphere samples. Heat map displays the sum read numbers of all samples for each genus. Data were transformed using the natural logarithm (log10).

For the microorganisms, we defined the 100 most abundant genera among all the samples as the core microbiomes of *C*. *communis*. According to Figure 5, the percentages of the total bacterial communities covered by the core microbiomes were 84.78% in the endosphere and 51.17% in the rhizosphere. The core microbiomes were classified into 10 bacterial phyla, and most of the genera in the core microbiomes belonged to the phyla Proteobacteria (42 genera), Bacteroidota (15 genera), Actinobacteriota (14 genera) and Firmicutes (14 genera), with the rest belonging to the phyla Desulfobacterota, Acidobacteriota, Chloroflexi, Nitrospirota, Gemmatimonadota and Bdellovibrionota.

### 3.7. Discriminative Taxon between the Endosphere and Rhizosphere Bacteria Community

**Figure 6:**
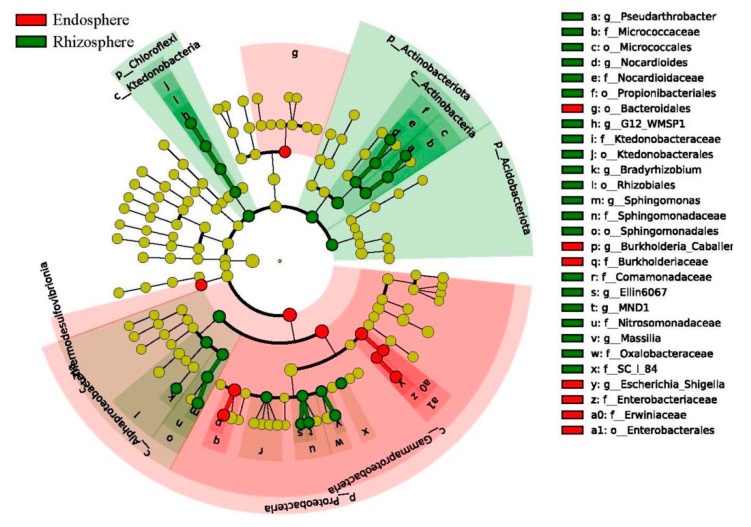
Cladograms, generated from LEfSe analysis, represent the polygenetic distribution of bacterial taxa. Green circles represent taxa that were significantly abundant in rhizosphere, while red circles represent taxa that were significantly abundant in endosphere. Yellow circles represent a non-significant changed taxon. The circles from inside to outside indicate bacterial taxonomic levels from phyla to genera, each circle’s diameter is proportional to the given taxon’s relative abundance.

The LEfSe analysis was used to identify discriminative taxon between the rhizosphere and endosphere communities in *C*. *communis*. An LDA score of 4.0 was used to identify bacterial groups with statistical significance. Twenty-seven of the taxa were in the rhizosphere bacterial communities of *C*. *communis* and ten of taxon exhibited significant differences in the endosphere communities (Figure 6 and Figure S7). Specifically, the most differentially abundant microbial taxa in endosphere communities were Proteobacteria (the phylum), Gammaproteobacteria (the class), Enterobacterales (the order) and Burkholderiaceae (the family). In contrast, Actinobacteriota (the phylum), Acidobacteriota (the phylum), Chloroflexi (the phylum), Alphaproteobacteria (the class), Actinobacteria (the class), Rhizobiales (the order), Sphingomonadales (the order), Sphingomonadaceae (the family), Nitrosomonadaceae (the family) and Comamonadaceae (the family) were significantly enriched in the rhizosphere communities. 

### 3.8. Relationships between Community Structure and Environmental Factors

**Figure 7:**
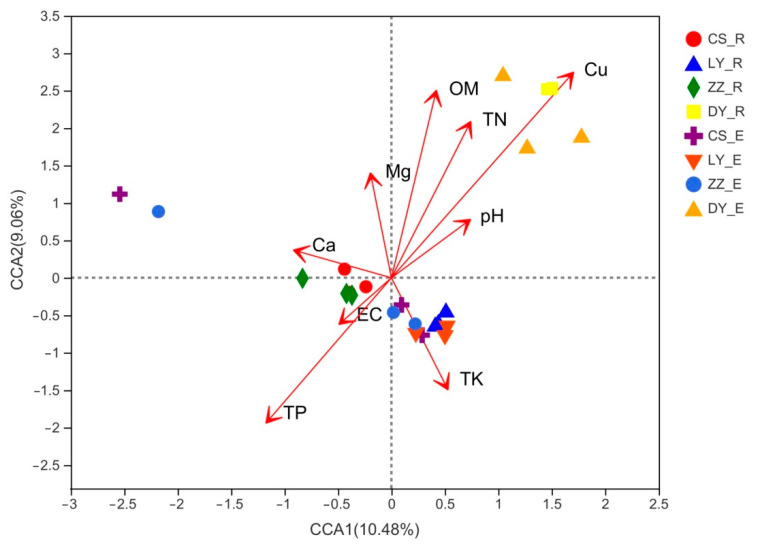
Canonical correspondence analysis (CCA) to show the correlations between the bacterial communities and soil physico-chemical properties. Arrows indicate the direction and magnitude of environmental parameters associated with bacterial community structure. R and E represent Rhizosphere and Endosphere, respectively.

The CCA was performed to discern the possible correlations of environmental variables with bacterial community structure. As shown in Figure 7, among the examined soil chemical properties, Cu (*R*^2^ = 0.977, *p* = 0.001), OM (*R*^2^ = 0.573, *p* = 0.002), TP (*R*^2^ = 0.466, *p* = 0.006) and TN (*R*^2^ = 0.437, *p* = 0.012) were the main soil environmental factors that significantly affected the bacterial community assembly and composition of *C*. *communis*. Pearson moment correlation analysis showed that the number of OTUs and Chao index for endophytic bacteria were all negatively correlated with the soil OM, TP, TN and Cu content (Table S3). The Shannon index was found to be negatively correlated with the OM, TN and Cu content (Table S3). Similarly, rhizosphere bacteria richness showed significantly negative correlation with the soil OM, TN and Cu content, while rhizosphere bacteria diversity was negatively correlated with Cu content and positively correlated with TP (Table S3).

### 3.9. Molecular Ecological Network of the Bacterial Communities 

**Figure 8:**
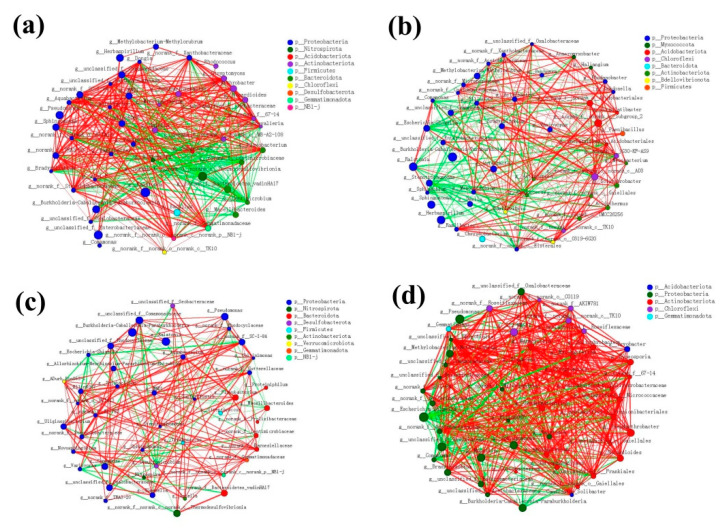
The networks of dominant microbiota at the genus level (top 50) in CS (**a**), LY (**b**), ZZ (**c**), and DY (**d**). The size of the nodes shows the abundance of OTUs, and the different colors indicate the corresponding taxonomic assignment at the phylum level. The edge color represents positive (red) and negative (green) correlations. The edge thickness indicates the correlation values; only significant interactions are shown (r > 0.6; *p* < 0.05).

A correlation network analysis at the genus level was conducted to further explore the complexity of the interactions within the bacterial communities at these four sites. As shown in Figure 8, the bacterial community of *C*. *communis* in the non-cupriferous site CS exhibited a higher level of complexity than that in the intermediate and low Cu contaminated sites. In an analysis of the top 50 bacteria at the genus level, there were 900 correlations in CS, 594 correlations in LY and 434 correlations in ZZ. It is worth noting that the microbial community network complexity is increased rather than decreased in the highly Cu-contaminated site DY (1104 correlations), indicting high Cu contamination does not significantly inhibit the microbial interspecific and intraspecific interaction. 

## 4. Discussion

Plants generally harbor vastly diverse microbiota in various plant compartments, including the rhizosphere, endosphere and phyllosphere, which contribute to the host plant’s health and productivity [35]. Although these plant microbiomes have widely explored in model plants (e.g., *Arabidopsis thaliana*) [36], crops (e.g., wheat, maize and) [37,38] and tress (e.g., poplar) [39], the information about the microbiomes associated with metallophytes is still very limited. Here, we systematically investigated the diversity and community structure of bacteria present in the rhizosphere and endosphere of *C*. *communis* growing on different Cu-polluted soils, which may provide prerequisite knowledge for Cu phytoremediation in the future. 

### 4.1. Variations of Bacterial Community Diversity and Structure between Rhizosphere and Endosphere 

Plant endophytes and rhizosphere bacteria constitute the microbial community of plant–micro–ecological environment [13]. In this study, we found that the parameters evaluating microbial diversity and richness were higher in the rhizosphere soil bacterial community than in the endophytic bacterial communities of *C*. *communis* (Figure 1 and Figure S3), which was consistent with the previous reports in other plant species [13,40,41]. The lower diversity and richness in the endosphere of *C*. *communis* can be explained partly by the environmental variability in different plant compartments [42]. The root rhizosphere is rich in carbon sources secreted by plants, thereby acting as chemical attractants and resulting in the formation of distinctive and diverse rhizosphere microorganisms [43,44]. Meanwhile, the rhizosphere acts as a bridge connecting plant roots and their surrounding soil environment, where complex chemical, physical, and biological interactions occur, including root–root, root–insect, and root–microbe interactions. These complex interactions need more bacteria with different functions, thus increasing the diversity and abundance of beneficial soil microorganisms [40]. Compared with the complicated rhizosphere areas, the relatively single environment inside the plant resulted in a relatively single microbial population. In addition, the microbial colonization and formation of stable communities in plant tissues must overcome the host plant’s innate immune system, which usually leads to a decrease in the density and diversity of the microbial community [45]. Thus, only a limited number of microbes could adapt to survive and/or proliferate in the endospheres of plants [46]. 

To further compare the bacterial community structure between the endosphere and rhizosphere of *C*. *communis*, all samples were clustered by the principal coordinate analysis (PCoA) and heat map in this study. Each plant compartment is a unique and heterogeneous microenvironment, thus leading to the formation of a set of specific plant-associated microbial communities [47,48]. Similar to previous studies, PCoA and hierarchical clustering results also showed that the rhizosphere and endosphere bacteria of *C*. *communis* were clearly distinguished according to their respective plant compartments (Figure 2). Furthermore, our data showed that the relative abundances of Actinobacteriota, Acidobacteria and Chloroflexi significantly decreased from the rhizosphere to the endosphere of *C*. *communis*. However, the relative abundance of Proteobacteria, Bacteroidota and Firmicutes was increased in the endosphere of *C*. *communis* compared with the rhizosphere (Figure 3a and Figure 5). These findings are consistent with studies that suggest plant compartment is a significant and strong driver for the differences in bacterial community structure [36,48,49,50].

### 4.2. Factors Influencing Bacterial Community Diversity and Composition

Heavy metal contamination in soils exerts toxicity against almost all microbes, thus leading to variations in microbial composition and diversity [51]. Although a trace amount of copper is necessary for the normal function of the ecosystem, while excess copper has toxic effects and been shown to be an important environmental factor shaping the composition and structure of bacterial communities [52]. In the present study, the CCA results revealed that the Cu content made the greatest contribution to variations in the soil bacterial community structure (*R^2^* = 0.977, *p* = 0.001) (Figure 7). Meanwhile, Cu content showed a negative association with the number of OTUs, Shannon index and Chao index in both endosphere and rhizosphere communities (Table S3). Generally, microbial diversity decreases as the contamination gradient increases, because an increase in toxic metal(loids) concentration generally means more harshness [53,54]. Similar to previous studies, the bacterial diversity and richness were found to be lowest in the samples collected from the heavily Cu contaminated site (DY) (Figure 1), indicating that a high level of Cu contamination could suppress or result in the death of sensitive soil microbes, and, therefore, subsequently affect the formation of the soil microbial community [55].

Bacterial communities not only depend on heavy metals, but are also affected by the soil properties such as pH, nutrients, and organic carbon content [56]. In this study, the CCA results demonstrated that the OM (*R*^2^ = 0.573, *p* = 0.002), TP (*R*^2^ = 0.466, *p* = 0.006) and TN (*R*^2^ = 0.437, *p* = 0.012) content were the main soil environmental factors, which significantly affected the assembly and composition of endophytic and rhizosphere bacterial community in the different *C*. *communis* samples (Figure 7). OM provides the carbon source for microorganisms and may significantly influence the biomass, activity, and composition of microbial communities [16,49,57]. Meanwhile, the increase in OM content can improve soil porosity, air circulation and water retention capacity, therefore promoting the growth of microorganisms [58]. Additionally, the nitrogen and phosphate are also the most important nutrients for microbial growth and affect the diversity and composition of soil bacterial communities [59]. Although soil pH has been reported to be a key factor influencing soil microbiomes [17,60], we did not find that there is a significant effect of soil pH on bacterial community structure in our study. 

Co-occurrence network can also provide profound and unique insights into microbial interactions and community assembly [61]. In our study, microbial communities in non-contaminated CS clearly exhibited a higher level of connectivity relative to on the community in Cu contaminated LY and ZZ (Figure 8). Interestingly, the bacterial community of *C*. *communis* in the severely contaminated site DY had a more complex network than that in intermediate and low Cu contaminated sites (Figure 8). In many cases, the levels of soil nutrients, such as the soil carbon and nitrogen contents, are the key factors related to shifts in the soil microbial community structure and network [62]. As shown in Table S1, the content of OM and TN in rhizospheric soil of *C*. *communis* in DY were significantly higher than those in other sites, which might cause changes in the relationships among the bacterial taxa [26,63]. Taken together, the results showed that both Cu contamination and soil properties were the main factors affecting the bacterial community structure and interaction in *C*. *communis*.

### 4.3. The Dominant Genera in the Endosphere and Rhizosphere of C. communis under High Level Cu Contamination

The detailed investigation for the specific groups was required to explore the possibility of changes in the bacterial community structure. In this study, we found *Sphingomonas* and *Pseudomonas* to be significantly more enriched in the rhizosphere and endosphere of *C*. *communis*, respectively, at the highly Cu-contaminated site DY (Figure 4). These two genera were heavy metal resistant and have been demonstrated as ecologically beneficial microorganisms, which could promote plant growth and facilitate phytoremediation [41]. Members of the genus *Sphingomonas* are Gram-negative, chemoheterotrophic and aerobic bacteria with plant-growth-promoting traits. For example, *Sphingomonas* sp. YM22 isolated from *C*. *communis* was found to possess multiple plant-growth-promoting characteristics, such as IAA, siderophores and ACC deaminase production and heavy metal-solubilizing ability [23]. Meanwhile, *Sphingomonas* sp. YM22 could be able to colonize rhizosphere soils of the maize plants and responsible for the protection of the maize plants against Cu toxicity in a heavily Cu-contaminated soil [64]. The strain *Sphingomonas SaMR12* was isolated from hyperaccumulator *Sedum alfredii*, which could promote plant growth and protect root of *S*. *alfredii* from Cd damage [65]. In another study, *Sphingomonas SaMR12* was reported to promote Cd root-to-shoot translocation and further shoot accumulation in a Cd accumulator *Brassica napus* [66]. *Pseudomonas* is also a well-known plant-associated bacterium, which can synthesis hormones to stimulate plant growth and increase resistance to various metals [67]. In previous studies, several *Pseudomonas* spp. have been isolated from heavy metal-rich environments. For example, Cu-tolerant *Pseudomonas putida* CZ1 acted as a plant-growth-promoting rhizobacterium, significantly enhanced the growth of the Cu-tolerant plant *Elsholtzia splendens*, and promoted the accumulation and translocation of Cu from roots to shoots [68]. *Pseudomonas koreensis* AGB-1, isolated from roots of *Miscanthus sinensis* growing in mine-tailing soil, exhibited high tolerance to Zn, Cd, As, and Pb [69]. Moreover, Babu et al. [69] found that inoculation *P*. *koreensis* AGB-1 promoted growth of *M*. *sinensis* by decreasing heavy metal toxicity through IAA and ACC deaminase production, which increased antioxidant enzyme activity and reduced lipid peroxidation. In short, members of these genera, markedly enriched in the rhizosphere and endosphere of *C*. *communis*, were promising bacteria for promoting plant growth and increasing the efficiency of phytoremediation in Cu-polluted soils [70].

## 5. Conclusions

This study provided in-depth analysis of endophytic and rhizosphere bacterial community profiles of a metallophytes *C*. *communis* growing in different Cu-polluted soils for the first time by high-throughput sequencing technology. We proved that the diversity and richness of endosphere microbiomes was much lower than those of the rhizosphere microbiomes in *C*. *communis*. Simultaneously, there was a clear difference in community structure between endosphere and rhizosphere microbiota, suggesting the niche-specific influence on bacterial communities. Among the environmental factors, soil Cu, OM, TP and TN content played major roles in shaping the bacterial community structure of *C*. *communis*. The core members of the endophytic and rhizospheric microbial communities in *C*. *communis* identified in this study could provide the basis for the isolation of specific functional bacterial strains and the field application of these strains in facilitating Cu phytoremediation efficiency.

## Data Availability

The raw sequencing data have been deposited in the NCBI Sequence Read Archive under BioProject accession code PRJNA698425.

## Supplementary Materials

The following are available online at https://www.mdpi.com/article/10.3390/microorganisms9081689/s1, Table S1: Physico-chemical properties of soil samples from the *C*. *communis* used in this study, Table S2: Summary of sequences obtained for the endosphere and rhizosphere of *C*. *communis*, Table S3: Pearson moment correlation analyses of environmental factors and bacterial diversity and abundance of endoshphere and rhizosphere of *C*. *communis*, Figure S1: Locations of all sampling sites for *C*. *communis* in this study, Figure S2: The rarefaction curve analysis of the bacterial sequences. The curves were constructed using the Shannon index values of the OTUs and the number of reads, Figure S3: The values of alpha diversity indexes (Shannon and Chao) in endosphere and rhizosphere microbial populations of *C*. *communis* at the four sampling sites (colorful dots) and their corresponding endosphere and rhizosphere means and 95% confidence intervals (solid black dots and vertical error bars, respectively). The significance levels of the difference between endosphere and rhizosphere means for each alpha metric resulting from ANOVA tests. *** indicates significant differences at *p* < 0.001, Figure S4: Principal coordinate analysis (PCoA) of bacterial communities in the endosphere (a) and rhizosphere (b) of *C*. *communis* at the four sampling sites, Figure S5: Venn diagrams of shared OTUs in endoshpere (a) and rhizosphere (b) of *C*. *communis* plants among the four sampling locations, Figure S6: Bacterial community composition at the phylum level in the endosphere and rhizosphere of *C*. *communis*, Figure S7: Enriched taxa of the bacterial communities reaching a linear discriminant analysis (LDA) significance threshold of 4.0.
