# Supplementary material for: Deciphering the Endophytic and Rhizospheric Microbial Communities of a Metallophyte Commelina communis in Different Cu-Polluted Soils
Source: Microorganisms. 2021 Aug 9;9(8):1689. doi: 10.3390/microorganisms9081689 (PMC8399850; doi:10.3390/microorganisms9081689)
Supplement: Supplementary file 1 [file microorganisms-09-01689-s001.zip › microorganisms-1318606-supplementary.pdf]

Supplementary Materials

Deciphering the Endophytic and Rhizospheric Microbial Communities of a Metallophyte *Commelina communis* in Different Cu-Polluted Soils

Li He [1,2,†], Yanzhen Ren [1,2,†], Weimin Zeng [1,2], Xueling Wu [1,2], Li Shen [1,2], Runlan Yu [1,2], Yuandong Liu [1,2] and Jiaokun Li [1,*]

[1] School of Minerals Processing and Bioengineering, Central South University, Changsha 410083, China; 195612132@csu.edu.cn (L.H.); 185612135@csu.edu.cn (Y.R.); zengweimin1024@126.com (W.Z.); wxlcsu@csu.edu.cn (X.W.); lishen@csu.edu.cn (L.S.); yrl715@sina.com (R.Y.); ydoliu@sina.cn (Y.L.)

[2] Key Laboratory of Biometallurgy, Ministry of Education, Central South University (CSU), Changsha 410083, China

* Correspondence: lijiaokun@csu.edu.cn

† These authors contributed equally to the work.

Table S1. Physicochemical properties of rhizosphere soil samples from the *C. communis* used in this study.

Sample	pH	OM (g/kg)	TN (g/kg)	TP (g/kg)	TK (g/kg)	EC (μS/cm)	Mg (mg/kg)	Ca (mg/kg)	Cu (mg/kg)
**CS**	6.26 ± 0.16a	11.52 ± 0.98a	0.84 ± 0.12a	1.55 ± 0.22a	16.7 ± 1.11a	70.96 ± 3.06a	324.33 ± 25.03a	1065.28 ± 15.02a	25 ± 3.15a
**LY**	7.37 ± 0.37b	3.24 ± 0.10b	0.23 ± 0.04b	1.35 ± 0.15ab	19.5 ± 1.32b	467.67 ± 27.23b	507.34 ± 20.03b	1211 ± 62.34a	41 ± 3.61b
**ZZ**	8.31 ± 0.15c	3.93 ± 0.72b	0.20 ± 0.03b	1.13 ± 0.14b	12.1 ± 0.51c	952 ± 30.12c	22100 ± 24.25c	26700 ± 182.81b	248 ± 28.91c
**DY**	8.16 ± 0.05c	16.7 ± 0.72c	1.23 ± 0.08c	0.74 ± 0.07c	13.7 ±0.41c	278 ± 10.01d	17500 ± 25.89d	7900 ±25.63c	13100 ± 132.28d

OM, organic matter; TC, total carbon; TN, total nitrogen; TP, total phosphorus; TK, total potassium; EC, electrical conductivity; The values represent means ± standard deviation (n = 3); Different small letters in a single column represent significant difference ($p < 0.05$).

Table S2 Summary of sequences obtained for the endosphere and rhizosphere of *C. communis*.

	Endoshpere					Rhizosphere				
	No. of sequences	No. of bases	Average length	No. of OTU	Coverage	No. of sequences	No. of bases	Average length	No. of OTU	Coverage
**CS_1**	60,812	22,833,330	375.47	458	99.88%	20,903	7,877,314	376.85	835	97.54%
**CS_2**	69,981	26,239,549	374.95	550	99.92%	24,993	9,416,729	376.77	946	97.19%
**CS_3**	65,373	24,562,782	375.47	686	99.62%	21,804	8,219,445	376.96	819	96.45%
**LY_1**	72,794	27,313,140	375.21	413	99.88%	24,402	9,146,945	374.84	641	98.83%
**LY_2**	74,207	27,811,044	374.78	567	99.87%	22,727	8,537,262	375.64	708	98.34%
**LY_3**	74,972	28,029,837	373.87	497	99.82%	20,841	7,832,972	375.84	765	98.00%
**ZZ_1**	69,476	26,072,764	375.28	760	99.80%	23,698	8,916,425	376.25	1085	96.92%
**ZZ_2**	68,034	25,438,611	373.91	562	99.79%	24,025	9,038,907	376.23	1086	96.28%
**ZZ_3**	66,999	25,112,162	374.81	656	99.82%	20,817	7,827,262	376.01	1060	96.25%
**DY_1**	23,271	8,746,496	375.85	167	99.98%	22,840	8,559,128	374.74	402	99.08%
**DY_2**	24,774	9,345,940	377.25	151	99.70%	18,987	7,108,758	374.40	402	99.01%
**DY_3**	18,966	7,148,983	376.94	45	99.91%	17,858	6,682,173	374.18	417	99.15%

Table S3 Pearson moment correlation analyses of environmental factors and bacterial diversity and abundance of endoshphere and rhizosphere of *C. communis*. * and ** indicate significant differences at $p < 0.05$ and 0.01, respectively.

Environmental factors	Pearson correlation coefficient					
	Endosphere			Rhizosphere		
	No. of OTUs	Shannon index	Chao index	No. of OTUs	Shannon index	Chao index
**pH**	-0.237	-0.232	-0.302	-0.098	-0.346	-0.088
**OM**	-0.708*	-0.645*	-0.731*	-0.674*	-0.500	-0.640*
**TN**	-0.707*	-0.678*	-0.741*	-0.659*	-0.483	-0.626*
**TP**	0.630*	0.551	0692*	0.523	0.642*	0.508
**TK**	0.159	0.093	0.221	-0.134	-0.020	-0.175
**EC**	0.417	0.343	0.389	0.554	0.282	0.553
**Mg**	-0.171	-0.175	-0.241	0.069	-0.130	0.105
**Ca**	0.308	0.250	0.250	0.536	0.312	0.561
**Cu**	-0.896**	-0.801**	-0.935**	-0.824**	-0.825**	-0.797**

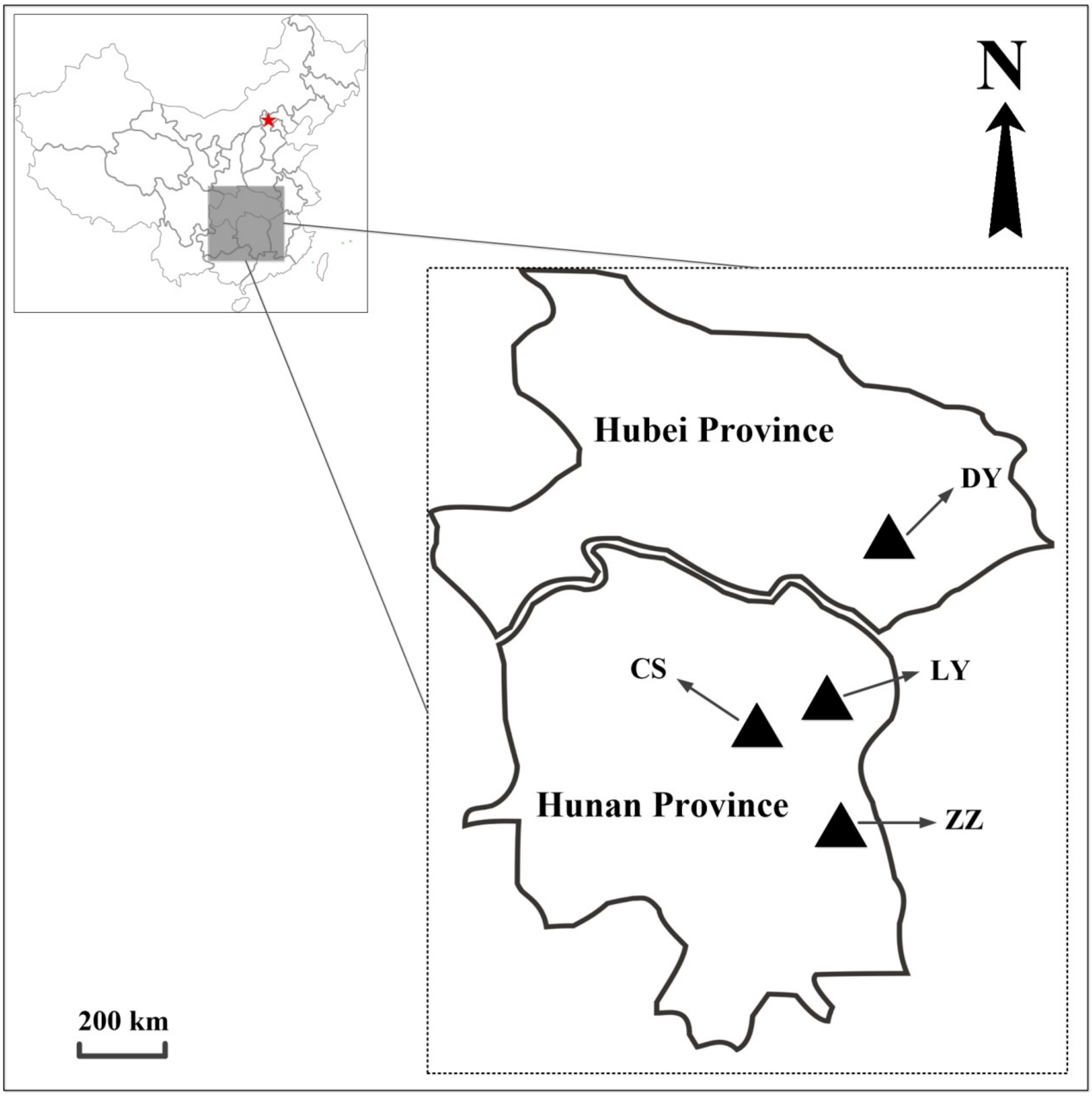

Figure S1. Locations of all sampling sites for *C. communis* in this study.

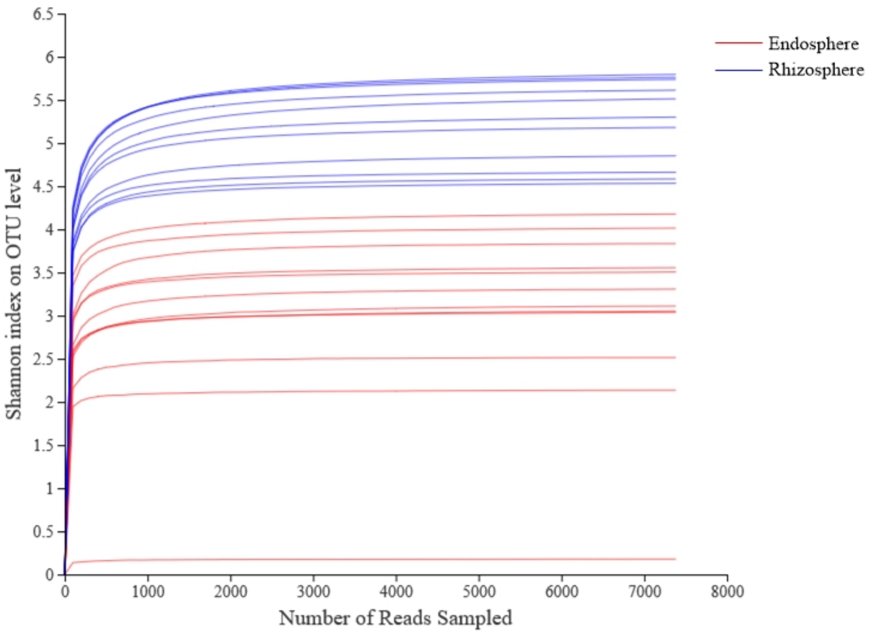

Figure S2. The rarefaction curve analysis of the bacterial sequences. The curves were constructed using the Shannon index values of the OTUs and the number of reads.

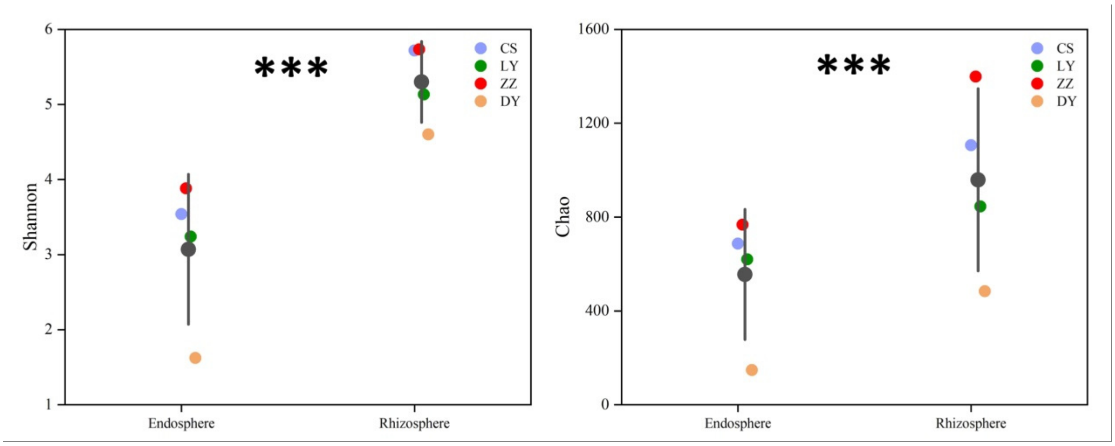

Figure S3. The values of alpha diversity indexes (Shannon and Chao) in endosphere and rhizosphere microbial populations of *C. communis* at the four sampling sites (colorful dots) and their corresponding endosphere and rhizosphere means and 95% confidence intervals (solid black dots and vertical error bars, respectively). The significance levels of the difference between endosphere and rhizosphere means for each alpha metric resulting from ANOVA tests. *** indicates significant differences at $p < 0.001$.

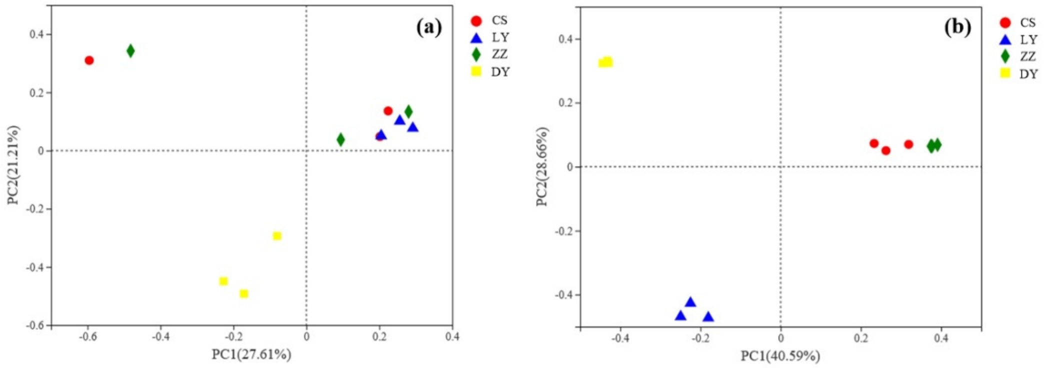

Figure S4. Principal coordinate analysis (PCoA) of bacterial communities in the endosphere (a) and rhizosphere (b) of *C. communis* at the four sampling sites.

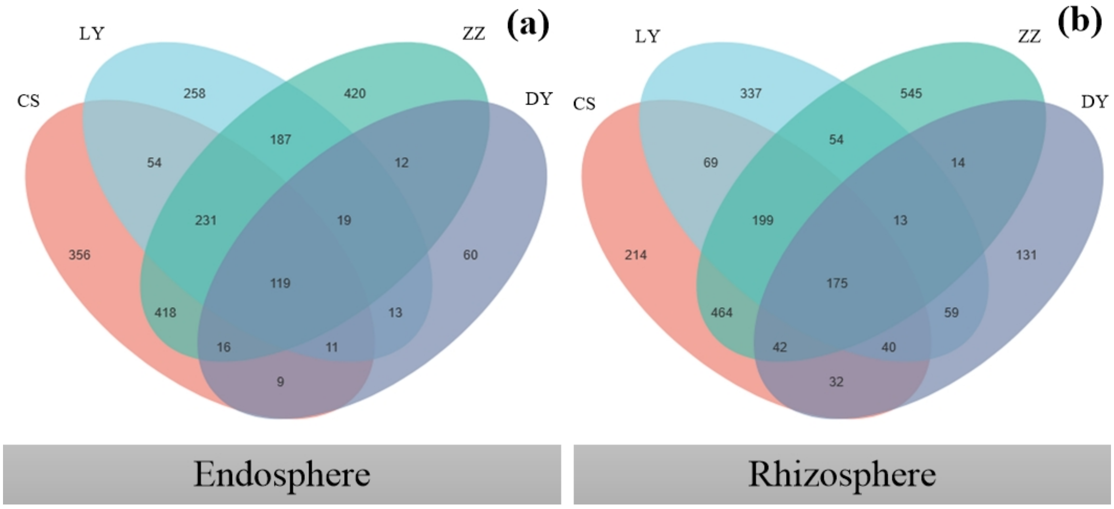

Figure S5. Venn diagrams of shared OTUs in endoshpere (a) and rhizosphere (b) of *C. communis* plants among the four sampling locations.

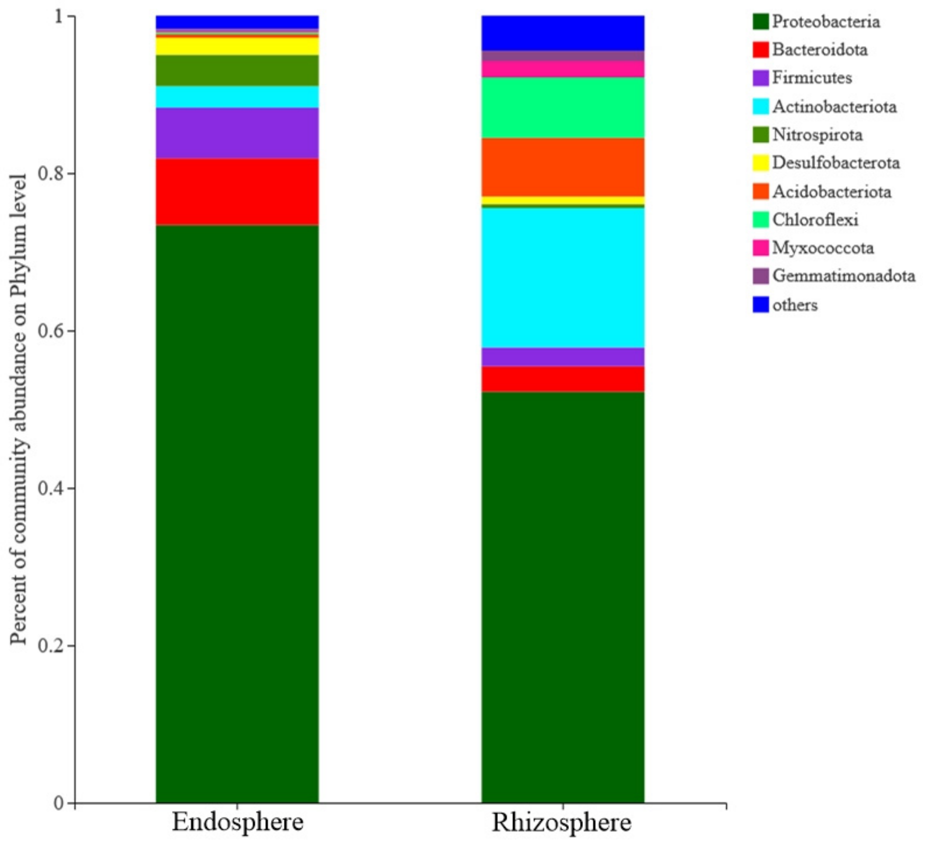

Figure S6. Bacterial community composition at the phylum level in the endosphere and rhizosphere of *C. communis*.

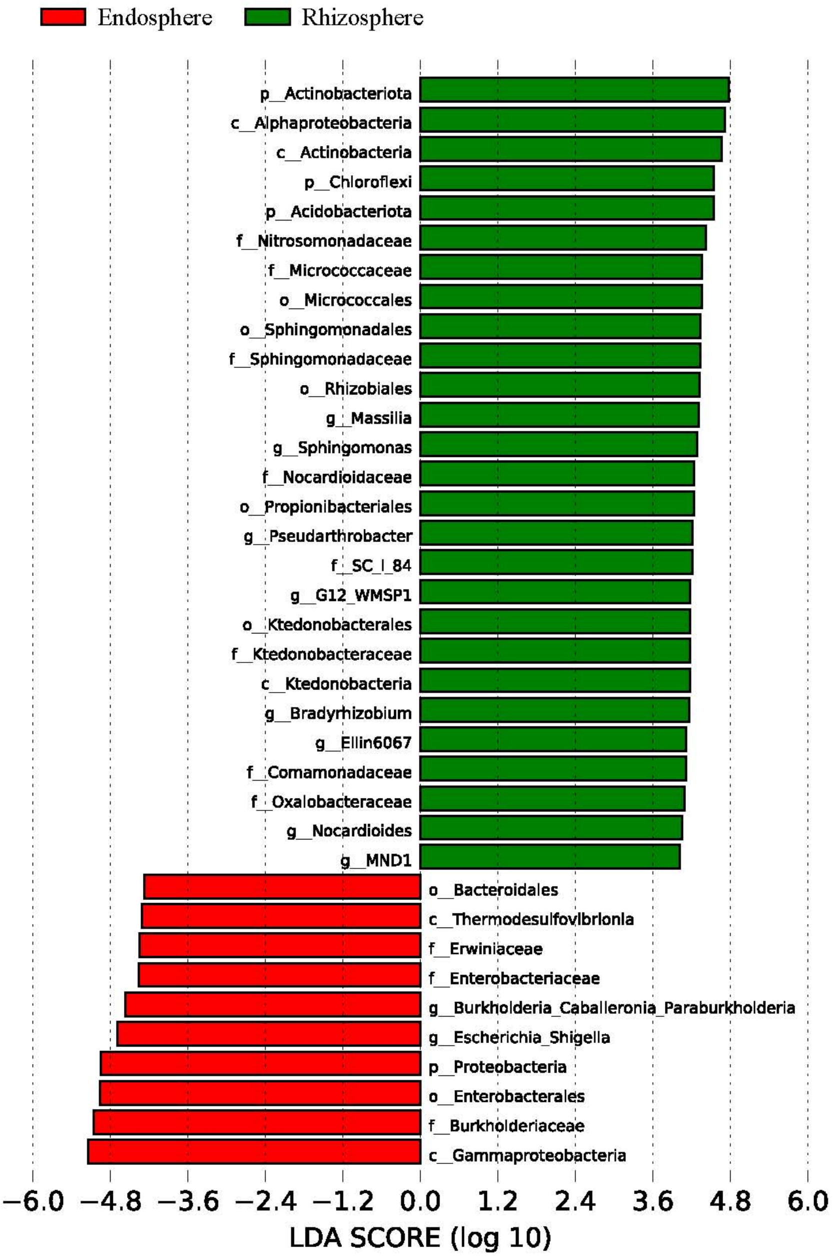

Figure S7. Enriched taxa of the bacterial communities reaching a linear discriminant analysis (LDA) significance threshold of 4.0.
